# A bibliometric analysis of malaria research in China during 2004–2014

**DOI:** 10.1186/s12936-015-0715-2

**Published:** 2015-05-10

**Authors:** Hang Fu, Tao Hu, Jingyi Wang, Da Feng, Haiqing Fang, Manli Wang, Shangfeng Tang, Fang Yuan, Zhanchun Feng

**Affiliations:** School of Medicine and Health Management, Tongji Medical College, Hua Zhong University of Science and Technology, No.13 of Hangkong Road, Qiaokou District, Wuhan, Hubei Province 430030 China; Bureau of Disease Prevention and Control of the National Health and Family Planning Commission of the People’s Republic of China, Beijing, China; School of Public Health, Wuhan University School of Medicine, Wuhan University, Wuhan, Hubei Province China

**Keywords:** Malaria imported malaria, Falciparum malaria, Vivax malaria, Malaria surveillance

## Abstract

**Background:**

China has made great progress in malaria prevention and control, but there has been no research to provide a macroscopic overview of malaria research in China. This bibliometric analysis was conducted from international databases to explore the characteristics of malaria investigations in China.

**Methods:**

Published scientific papers about malaria were retrieved from China National Knowledge Infrastructure (CNKI), Wanfang database, Cqvip and PubMed during 2004–2014. Year of publication, first-author affiliation, journal name and keywords were extracted with the Bibliographic Items Co-occurrence Matrix Builder (BICOMB). High-frequency keywords were selected to construct the co-word matrix and divided into eight categories. Sub-networks were utilized to analyse the complex knowledge structures.

**Results:**

In recent ten years, a total of 5,126 entries were included. The number of papers on malaria started to increase since 2010. The papers published by top 12 Chinese journals in the field of malaria accounted for 32.98% in overall articles. Most of the studies were conducted by the researchers from the Centers for Disease Control and Prevention (CDCs). The words “malaria”, “imported malaria”, “falciparum malaria”, “vivax malaria” and “malaria surveillance” were the centers of knowledge structures.

**Conclusion:**

Chinese studies on malaria mainly focus on the epidemiology and biomedical fields, this study offers a systematic evaluation on the output of malaria studies and the elimination of malaria in China.

## Background

Malaria is a mosquito-borne infectious parasitic disease that is transmitted to people through the bites of Anopheles mosquitoes. In 2013, malaria was considered endemic in 104 countries and territories [1], including China. Several nationwide outbreaks of malaria have occurred in Chinese history, and with the unprecedented governmental and international organizational efforts, malaria cases has been dramatically decreased from thirty million (reported in 1949) to 2,460 (data as of September 2014) [2, 3]. Especially in recent 10 years, the malaria epidemic situation has been effectively controlled, and China has moved from a stage of malaria control to a stage of malaria elimination [4]. However, some problems also emerged, including the increasing number of foreign imported malaria cases and the persistence of malaria surveillance after elimination, which bring new challenges to malaria elimination work in China [5].

Bibliometrics, a well-established research method in information science [6], has been used in malaria vaccines and malaria in pregnancy [7, 8]. In addition, Garg et al. analysed the worldwide malaria research output during 1990–2000 [9]. Gupta and Bala estimated the research output of Indian malaria research in both national and global context during 1998–2009 [10]. In China, although there were systematic reviews on congenital malaria cases and malaria outbreaks (1990–2013) [11, 12], few studies have been performed to provide a macroscopic overview of malaria research by using the bibliometric method, probably due to language barriers and poor compatibility between Chinese databases and international bibliometric softwares. Here, the literature from both English and Chinese databases were searched, and the output of malaria research within the recent decade (2004–2014) was summarized by employing Bibliographic Items Co-occurrence Matrix Builder (BICOMB) software.

## Methods

### Date resource

China National Knowledge Infrastructure (CNKI) is the world’s largest Chinese journal full-text database, and has abundant literature resources as well as good retrieval functions. Wanfang database, Cqvip and PubMed were also considered in this study. The word “malaria” was used as a subject, and 6,818 records were found within these online databases with the inclusion dates from 1 January 2004 to 31 December 2014. After excluding the publications as newspapers, profiles, letters, editorials as well as other irrelevant literatures, a total of 5,126 records were eventually included.

### Information extraction

Bibliographic Items Co-occurrence Matrix Builder (BICOMB) was designed to accurately extract and count the bibliographic information from worldwide databases to generate the co-occurrence matrix and provide basic data for subsequent statistical analysis. Relevant articles can be retrieved via the publishing year, journal, affiliations of first author and keywords by using BICOMB. BICOMB was supported by China Health Policy Support Project and further upgraded to version 2.0 funded by China Medical University.

In the process of keyword extraction, the process of eliminating irrelevant data was required. The numbers and letters which were irrelevant keywords were removed because of the format problem in Chinese literatures. Since Chinese words had a variety of synonyms, similar words had to be semantically integrated, such as “migrant workers”, “overseas workers” and “returned labourers” into one single word “migrant workers”. This process was necessary for subsequent keyword co-occurrence analysis.

### Data analysis

Year of publication, journals, and affiliations of articles with high frequency, were counted and top ten literatures by citation frequency in the field of malaria were automatically computed by BICOMB. For the keywords, high-frequency keywords were defined as those words with a frequency of ≥ 55 times. All keywords were classified according to the connotations and features. Co-word analysis was also utilized and co-word matrix was created by BICOMB and then input to the Ucinet 6 software for social network analysis.

## Results

### Number of malaria-related publications

**Fig. 1:**
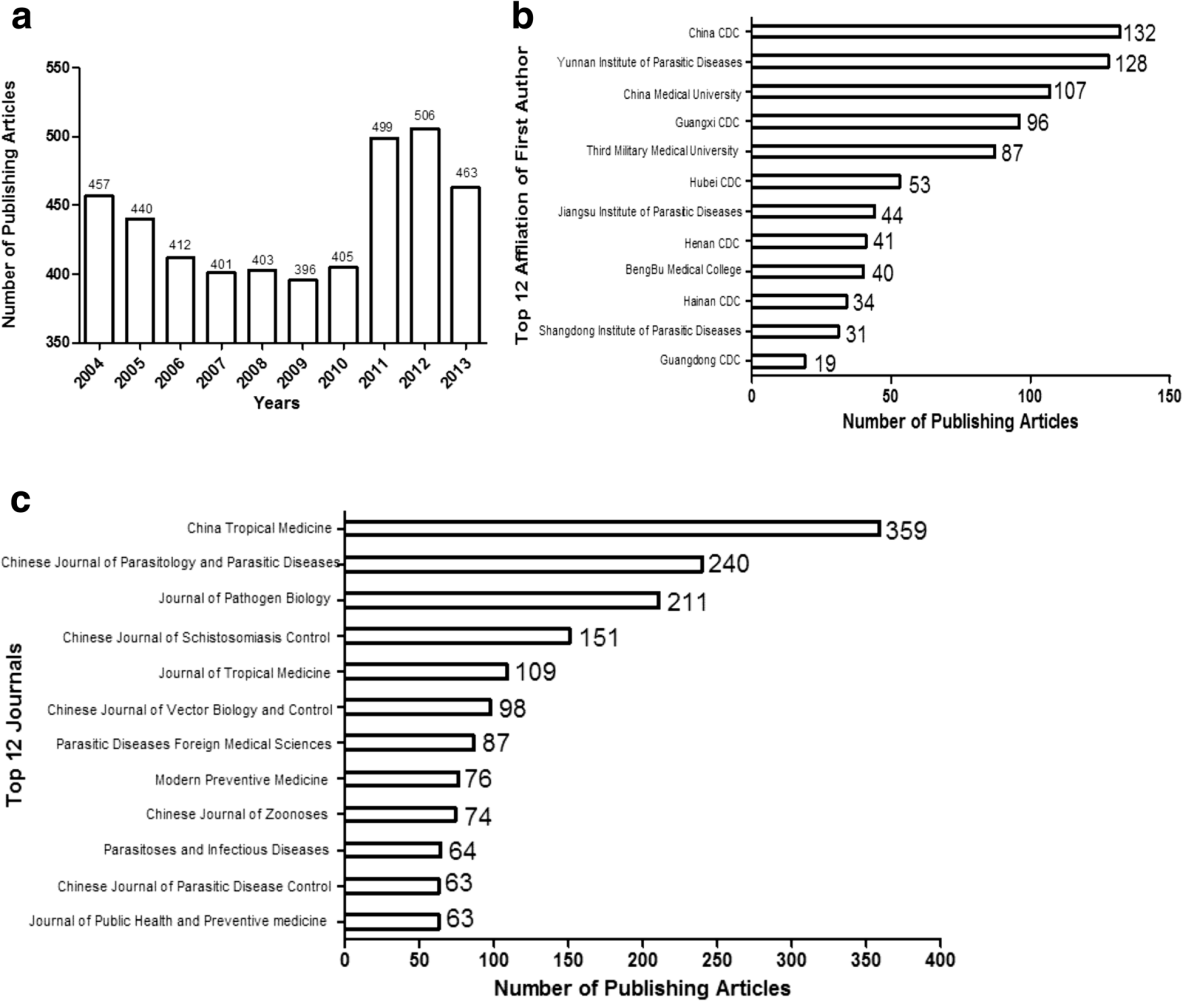
Characteristics of published articles in malaria research of China. The number of publishing articles during the period of 2004–2014 is shown in (1**a**), of the top 12 affiliations is illustrated in (1**b**) and of the top 12 journals is shown in (1**c**)

The quantity of malaria papers was 457 in 2004 and then decreased to 396 in 2009, increased in 2010, peaked in the year of 2012 with 506 papers and then declined to 463 in 2013 (Fig. 1a). Top-ranking 12 affiliations included six CDCs, two institutes of parasitic diseases (IPDs) and two universities (Fig. 1b). Most malaria-related papers were contributed by the researchers from CDC in China and IPD of Yunnan province. Top-ranking 12 journals in the field of malaria were shown in Fig. 1c, accounting for 32.98% of the overall malaria studies focusing upon the malaria situation in China. In addition, the number of published literatures in the top four journals accounted for approximately 20% of all investigations.

### High-frequent citation articles

As shown in Table 1, the articles with relatively high citations within CNKI, Wanfang database, Cqvip and PubMed were listed. There were seven articles describing the malaria situation in China written by Shuisen Zhou et al. from CDC in China [13-19]. After removing these seven articles, the top 10 articles assessed by citation frequency were listed in the right column of Table 1. In the most frequently cited article, autoregressive integrated moving average (ARIMA) model was adopted to predict the incidence of malaria [20]. The 2^nd^ and 7^th^ articles described the current situation of malaria prevention and control, as well as the opportunities and challenges of fighting against malaria in China [21, 22]. The 4^th^, 5^th^, 7^th^ and 9^th^ papers analysed the malaria situation in Jiangsu and Anhui provinces, China [23-26]. Malaria situation in Southeast Asia was also introduced in the 10^th^ article [27]. The 3^rd^ article reviewed the anti-malarial effect and other major biological effects of artemisinin [28]. The impact of global warming upon transmission of malaria was studied in the 6^th^ paper [29].

**Table 1:** Ten articles with the highest citation frequency with malaria as main keyword

	Authors	Title	Citation	*	Authors	Title	Citation
1	Shuisen Zhou et al. 2011 [13]	Malaria Situation in the People’s Republic of China in 2010	71	1	Liang Wen et al. 2004 [20]	Prediction of malaria incidence in malaria epidemic area with time series models	63
2	Shuisen Zhou et al. 2007 [14]	Malaria Situation in the People’s Republic of China in 2006	67	2	Qi Gao, 2011 [21]	Opportunities and challenges of malaria elimination in China	52
3	Shuisen Zhou et al. 2006 [15]	Malaria Situation in the People’s Republic of China in 2005	66	3	Yan Guo et al. 2006 [28]	Recent advancement in pharmacological effects of artemisinin and its derivatives	39
4	Liang Wen et al. 2004 [20]	Prediction of malaria incidence in malaria epidemic area with time series models	63	4	Xiaolin Jin et al. 2006 [23]	Current epidemic status and influencing factors of malaria in Jiangsu Province	38
5	Shuisen Zhou et al. 2008 [16]	Malaria Situation in the People’s Republic of China in 2007	55	5	Huayun Zhou et al. 2009 [24]	Epidemic and control of malaria in Jiangsu Province	38
6	Qi Gao, 2011 [21]	Opportunities and challenges of malaria elimination in China	52	6	Kun Yang et al. 2006 [29]	Impact of global warming on transmission of vector-borne diseases in China	37
7	Shuisen Zhou et al. 2009 [17]	Malaria Situation in the People’s Republic of China in 2008	52	8	Liping Wang et al.2008 [26]	Spatial-temporal analysis on the distribution of malaria in Anhui, 1990-2006	36
8	Yan Guo et al. 2006 [28]	Recent advancement in pharmacological effects of artemisinin and its derivatives	39	7	Xingyi Zhang. 2008 [22]	Malaria situation and prevention and control in China	30
9	Zhigui Xia et al. 2012 [18]	Malaria Situation in the People’s Republic of China in 2011	38	9	Yunzu Shen 2006 [25]	Investigation on transmission factors of malaria in Anopheles sinensis areas in Anhui Province	30
10	Shuisen Zhou et al. 2005 [19]	Malaria Situation in the People’s Republic of China in 2004	38	10	Rilang Meng et al. 2010 [27]	Prevalence and control of malaria in countries of Southeast Asia	29

*Ten articles with the highest citation frequency after removing articles written by Zhou et al. in the left column

### Keyword grouping

In total, 44 keywords were classified into seven categories according to their connotations and features (Table 2). “Falciparum malaria”, “vivax malaria”, “cerebral malaria” and “malariae malaria” were assigned into the “malaria” group, and “imported malaria”, “floating population”, “migrant workers” and “imported falciparum malaria” were allocated into another category. The “plasmodium”, “vector”, “epidemiology”, “diagnosis and treatment” and “prevention and control” groups were also established. The number of keywords in “epidemiology” and “diagnosis and treatment” accounted for 56.82 % among all seven groups.

**Table 2:** Category of high frequency keywords

	Groups	High frequency keywords
1	Malaria	1. malaria (1374)*, 2. falciparum malaria (519), 6. vivax malaria (292), 29. cerebral malaria (94), 40. malariae malaria (58)
2	Plasmodium	4. *P. falciparum* (353), 8. Plasmodium (243), 18. *P. vivax* (139), 32. *P. yoelii* (88)
3	Vectors	16. *An. sinensis* (148), 28. *An. anthropophagus* (94)
4	Imported malaria	3. imported malaria (401), 15. floating population (151), 36. migrant workers (72), 43. imported falciparum malaria (56)
5	Epidemiology	5. malaria surveillance (322), 9. malaria epidemic (232), 13. epidemic situation (185), 14. malaria-endemic areas (185), 17. analysis of epidemic situation (140), 20. malaria incidence rates (133), 23. epidemiology (117), 24. reported malaria cases (109), 25. malaria transmission (105), 33. epidemiological traits (86), 37. epidemiological survey (69), 41. epidemic trend (57), 42. Investigation (57)
6	Diagnosis and Treatment	7. antimalarial medicines (278), 10. artemisinin (214), 11. diagnosis and treatment (205), 19. malaria patients (137), 21. malaria vaccines (131) 22. primaquine (127), 27. artemether (94), 31. infectious disease (88), 34. artesunate (84), 35. blood film (77), 38. insecticide resistance (65), 39. plasmodium infection (60)
7	Prevention and Control	12. preventative and curative measures (190), 26. prevention and control strategies (102), 30. prevention and control effects (88), 44. Global Fund (54)

*The serial numbers in front of keywords are the rankings of occurrence frequency, and the numbers in parentheses are the frequency of key words

### Knowledge structure

Co-occurrence of words in the same literature reflects the relevance of the themes, and the keywords network generated by co-words matrixes can reflect the knowledge structure [30]. In the network of this article, the mean value of betweenness centrality of the keywords is 5.750 ± 6.300, the minimum value is 0.000, the maximum value is 27.814, and the network centralization index is 1.25 %. Due to the complexity of the overall network, the complete network graph was not shown in this paper. Instead, a set of sub-networks constructed by different inclusive criterion of co-occurrence frequency were illustrated for reference.

**Fig. 2:**
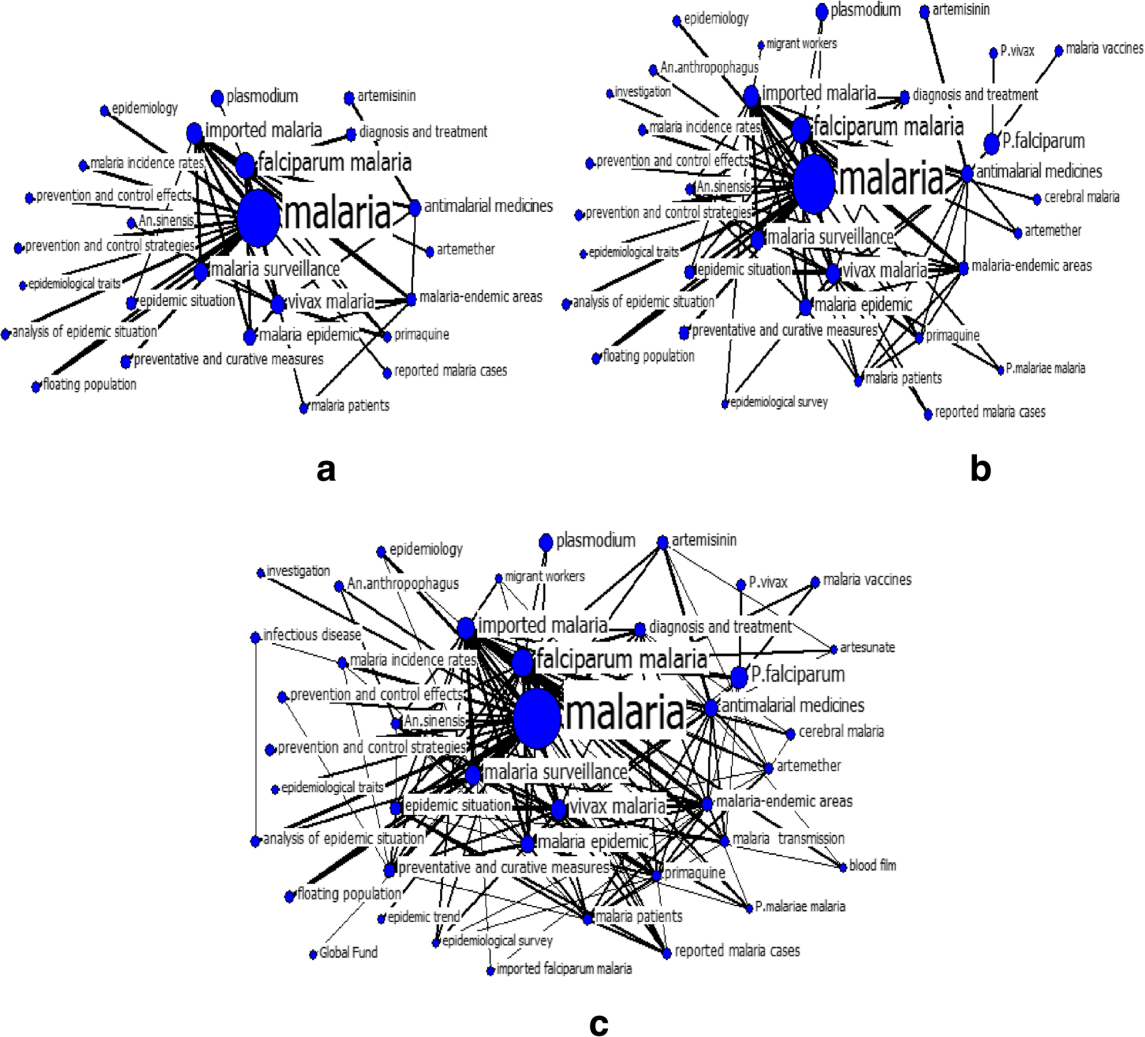
Sub-networks for keywords in malaria research of China, 2004–2014. *The size of nodes indicates the keywords centrality, and the thickness of the lines indicates the co-occurrence frequency of keywords pairs. **a** represents the keyword pairs with a co-occurrence frequency ≥45, **b** denotes ≥30 and **c** represents ≥15

The overall structure of malaria research in China when the threshold was 45 was shown in Fig. 2a. Keywords including “malaria”, “imported malaria” and “falciparum malaria” possessed the largest betweenness centrality and played a pivotal role in the network. “Plasmodium”, “anti-malarial medicines”, “vivax malaria”, “malaria surveillance” and “malaria epidemic” were equally regarded as the main keywords. Along with the threshold declining from 45 to 15, the link among existing keywords became denser. As new keywords were supplemented, the link among original words was closer and the central role of other keywords gradually highlighted. “*P. falciparum*”, not shown in Fig. 2a, was linked with “*P. vivax*”, “anti-malarial drugs”, “malaria vaccines” in Fig. 2b, and had new co-occurrence relationship with “imported malaria”, “falciparum malaria” and “malaria transmission” in Fig. 2c, suggesting that anti-malarial drugs and vaccine against falciparum malaria and the transmission of imported falciparum malaria served as vital parts of malaria investigations in China.

In the network shown in Fig. 2, both the sub-section of the research and the degree of correlation among keywords were represented. The thick lines connecting two keywords, such as “artemisinin” and “anti-malarial medicines”, “vivax malaria” and “primaquine”, denoted that these keywords had a higher co-occurrence rate and were more significantly correlated.

## Discussion

The number of articles began to increase in 2010, and the most important reason for that is the “Action Plan of China Malaria Elimination (2010–2020)” was initiated in this year, when the profile of malaria in China has changed significantly. The prevalence of malaria has declined dramatically as a result of this malaria elimination programme. There was a total decline of 34,320 malaria cases between 2006 and 2012 with a mean annual incidence of 0.04 per 10,000 by 2012. Meantime, the number of counties reporting autochthonous cases declined from 290 to 19. From a geographic point of view, five provinces in central China have successfully reduced the burden of malaria due to *Plasmodium vivax* in the last decade. More work has been done and better performance has been obtained in Anhui and Jiangsu provinces compared with their counterparts.

The sustained success of the programme depends upon the accurate laboratory diagnosis of malaria. The fundamental requirements are a reliable malaria diagnosis laboratory network, quality management system to validate case verification and source tracking. Besides the annual report of the malaria situation, the main purpose of malaria research also includes the prediction of malaria, the anti-malarial medicines and the impact of climate factors. The epidemiological changes in the number of autochthonous cases over time were examined to discuss the feasibility of achieving the goal of malaria elimination by 2020.

According to BICOMB analysis, CDCs and IPDs in China are the major affiliations publishing the most malaria-related papers because these institutions play a central role in the prevention and control of malaria nationwide and accumulate abundant laboratory and clinical experiences. Based upon the frequently-cited articles from multiple databases, most studies were conducted by the researchers from CDC or its affiliated units. However, another severe flaw is emerging. Independent third party is not involved in current malaria-related investigations. Hence, independent universities and other social organizations should actively participate in the relevant research, aiming to eliminate the study bias.

Keywords not only function to search, but also serve as signal words reflecting the theme, characteristics and trend in certain filed. Keyword classification revealed the following features: (1) “Falciparum malaria”, “vivax malaria”, “cerebral malaria”, “malariae malaria” and “imported malaria” are major types of malaria in Chinese malaria-related studies. (2) Besides *P. falciparum* and *P. vivax*, studies on *Plasmodium yoelii* are also widely performed in China. (3) *Anopheles sinensis* and *Anopheles anthropophagus* are the two main vectors of malaria in China. (4) Epidemiological research is the foundation and main part of malaria investigations in China. (5) The use of anti-malarial drugs and malaria vaccines is regarded as specific measures for the prevention and control of malaria, and is becoming a research focus in China. (6) “Global Fund” is a unique keyword among high-frequency keyword pool, highlighting the pivotal role of the Global Fund in the prevention and treatment of malaria in China. The microscope equipments, personnel training, knowledge education, monitoring source of infection and alternative aspects have been significantly enhanced, and thus the therapeutic effect of malaria prevention and treatment has been remarkably elevated with financial support from the Global Fund [31]. In addition, the underlying mechanism of supervision and evaluation, as well as the ideas of scientific management in the Global Fund Malaria Project provide meaningful references for Chinese practitioners [32].

Network map represents that the epidemiological studies are the dominant part of malaria investigations, which is consistent with the findings of keyword classification. The main knowledge structure reveals that imported malaria is becoming an emphasis of malaria research in China during the past decade, probably related to the promotion of preventative measures and national socio-economic development [33]. At present, indigenous malaria cases in China are gradually being eliminated. No malaria cases have been reported in Jiangsu province for three consecutive years since September 2011. Imported malaria, especially imported falciparum malaria, has become a novel challenge and thus surveillance and management of migrant workers departing from malaria epidemic countries is of vital significance. Therefore, it is necessary to establish a joint mechanism of malaria prevention and control among CDCs, medical institutions, inspection and quarantine institutions and other departments. Moreover, health education, drug prevention, personnel and technology training, follow-up supervision, are urgently needed to protect the physical health of returning workers [34,35]. Malaria surveillance including the surveillance of imported malaria is the main task during the later stage of malaria elimination programme [21]. In addition, along with the alleviation in malaria epidemic situation, how to maintain the alerted attitude of medical staff and guarantee the quality and efficiency of malaria surveillance remain to be urgently and properly resolved.

## Conclusion

Previous malaria-related studies have mainly focused on epidemiological and biomedical aspects, this investigation attempts to conduct a systematic evaluation and analysis on the elimination of malaria from the perspective of the management and policies in the late stage of malaria elimination, aiming to offer evidence and reference for both China and other countries.

### Study limitations

First, this study merely covers major scientific databases but fails to carry out the bibliometric analysis of small-scale databases or university libraries. Second, some Chinese articles lack specific language and format, which influences to a certain extent subsequent data processing.

## References

[1] WHO. World Malaria Report 2013. Geneva: World Heath Organization. Available: [http://www.who.int/malaria/publications/world_malaria_report_2013/en/].

[2] China Disease Prevention and Control Center. Report of notifiable infectious diseases in China*.* 2014. Available: [http://www.chinacdc.cn/tjsj/fdcrbbg/].

[3] Yip K (1998). Antimalarial work in China: a historical perspective. Parasitologia.

[4] Cao J, Zhou SS, Zhou HY, Yu XB, Tang LH, Gao Q (2013). Malaria from control to elimination in China: Transition of goal, strategy and interwentions] (in Chinese). Chin J Schistosomiasis Control.

[5] Feng J, Yan H, Feng XY, Zhang L, Li M, Xia ZG (2014). Imported malaria in China, 2012. Emerg Infect Dis.

[6] Jia X, Dai T, Guo X (2013). Comprehensive exploration of urban health by bibliometric analysis: 35 years and 11,299 articles. Scientometrics.

[7] Garg KC, Kumar S, Madhavi Y, Bahl M (2009). Bibliometrics of global malaria vaccine research. Health Info Libr J.

[8] van Eijk AM, Hill J, Povall S, Reynolds A, Wong HL, Ter Kuile FO (2012). The malaria in pregnancy library: a bibliometric review. Malar J.

[9] Garg KC, Dutt B, Kumar S, (2006). A preliminary scientometrics investigation of malaria research. Ann Libr Inf Stud.

[10] Gupta BM, Bala A (2011). A bibliometric analysis of malaria research in India during 1998–2009. J Vector Borne Dis.

[11] Tao ZY, Fang Q, Liu X, Culleton R, Tao L, Xia H (2014). Congenital malaria in China. PLoS Negl Trop Dis.

[12] Lu GY, Zhou SS, Horstick O, Wang X, Liu YL, Muller O (2014). Malaria outbreaks in China (1990–2013): a systematic review. Malar J.

[13] Zhou SS, Wang Y, Li Y (2011). Malaria Situation in the People’s Repulic of China in 2010] (in Chinese). Chin J Parasitol Parasit Dis.

[14] Zhou SS, Wang Y, Tang LH (2007). Malaria Situation in the People’s Repulic of China in 2006] (in Chinese). Chin J Parasitol Parasit Dis.

[15] Zhou SS, Wang Y, Tang LH (2006). Malaria Situation in the People’s Repulic of China in 2005] (in Chinese). Chin J Parasitol Parasit Dis.

[16] Zhou SS, Wang Y, Wen F, Tang LH (2008). Malaria Situation in the People’s Repulic of China in 2007] (in Chinese). Chin J Parasitol Parasit Dis.

[17] Zhou SS, Wang Y, Wen F, Tang LH (2009). Malaria situation in the People’s repulic of china in 2008] (in Chinese). Chin J Parasitol Parasit Dis.

[18] Xia ZG, Yang MN, Zhou SS (2012). Malaria situation in the People’s repulic of china in 2011] (in Chinese). Chin J Parasitol Parasit Dis.

[19] Zhou SS, Tang LH, Sheng HF, Wang Y (2005). Malaria situation in the People’s repulic of china in 2004] (in Chinese). Chin J Parasitol Parasit Dis.

[20] Wen L, Xu DZ, Lin MH, Xia JL, Zhang ZY (2004). Prediction of malaria incidence in malaria epidemic area with time series models] (in Chinese). J Fourth Mil Med Univ.

[21] Gao Q (2011). [Opportunities and challenges of malaria elimination in China] (in Chinese). Chin J Schisto Control.

[22] Zhang XY (2008). [Malaria situation and prevention and control in China] (in Chinese). Applied Prev Med.

[23] Jin XL, Gao Q, Zhou HY, Wang WM, Li JL, Gu YP (2006). [Current epidemic status and influencing factors of malaria in Jiangsu Province] (in Chinese). Chin J Schistosomiasis Control.

[24] Zhou HY, Cao J, Wang WM, Li JL, Gu YP, Zhu GD (2009). [Epidemic and control of malaria in Jiangsu Province] (in Chinese). Chin J Schistosomiasis Control.

[25] Shen LZ (2006). Investigation on transmission factors of malaria in Anopheles sinensis areas in Anhui Province (in Chinese). J Pathog Biol.

[26] Wang LP, Xu YF, Wang JJ, Xu X, Zhang WY, Fang LQ (2008). [Spatial-temporal analysis on the distribution of malaria in Anhui, 1990–2006] (in Chinese). Chin J Dis Control Prev.

[27] Meng RL, Huang YM (2010). [Prevalence and control of malaria in countries of Southeast Asia] (in Chinese). China Trop Med.

[28] Guo Y, Wang J, Chen ZT (2006). [Recent advancement in pharmacological effects of artemisinin and its derivatives] (in Chinese). Chin J Clin Pharmacol Ther.

[29] Yang K, Wang XH, Lv S, Zhang L, Jia TW, Li LH (2006). [Impact of global warming on transmission of vector-borne diseases in China] (in Chinese). Int J Med Parasit Dis.

[30] Morita K, Atlam E-S, Fuketra M, Tsuda K, Oono M, Aoe J-i (2004). Word classification and hierarchy using co-occurrence word information. Inf Process Manage.

[31] Xion L, Hu SJ, Xia GH, Zhang ZX, Yang HL, Zhou HN (2009). Analysis of effects of global malaria control program in Yunnan province] (in Chinese). J Pathog Biol.

[32] Li L, Mao SL, Xiao N, Xu GJ, Lei Y, Zhang RJ (2009). [Discussion on pattern of global fund malaria control project in Sichuan province] (in Chinese). J Prev Med Inf.

[33] Wang SQ, Li YC, Zhang ZM, Wang GZ, Hu XM, Qualls WA (2014). Prevention measures and socio-economic development result in a decrease in malaria in Hainan. China Malar J.

[34] Liu YB, Hsiang MS, Zhou HY, Wang WM, Cao YY, Gosling RD (2014). Malaria in overseas labourers returning to China: an analysis of imported malaria in Jiangsu Province, 2001–2011. Malar J.

[35] Dai J, Hong Y, Zhang XG, Zhang W, Deng J, Wu HM (2012). [The analysis of surveillance status and prevention measures on imported malaria cases at Guangdong Ports, 2010–2011] (in Chinese). Chin J Dis Control Prev.

